# Qualitative Content Analysis of COVID-19’s Role in Suicide Attempts Leading to Hospital Care

**DOI:** 10.3390/ijerph22121840

**Published:** 2025-12-09

**Authors:** Martina Mravlja, Anthony Pisani, Annamarie Bailey, Nicola Meda, Alexandre Paim-Diaz, Kristina Zurich, Kenneth Conner

**Affiliations:** 1 Slovene Centre for Suicide Research, Andrej Marušič Institute, University of Primorska, Muzejski trg 2, 6000 Koper, Slovenia; 2 Department of Pediatrics, University of Rochester Medical Center, 300 Crittenden Blvd., Rochester, NY 14642, USA; 3 Department of Psychiatry, University of Rochester Medical Center, 300 Crittenden Blvd., Rochester, NY 14642, USA; 4 Department of Neuroscience, University of Padua, Via Belzoni 160, 35121 Padova, Italy; nicola.meda@studenti.unipd.it; 5 Department of Psychiatry, University of Pittsburgh, 3811 O’Hara St., Pittsburgh, PA 15213, USA; 6 Lived Experience Consultants, Rochester, NY 14607, USA; 7 Departments of Emergency Medicine and Psychiatry, University of Rochester Medical Center, 300 Crittenden Blvd., Rochester, NY 14642, USA

**Keywords:** suicide, COVID-19, pandemic mental health, loneliness and isolation, healthcare, stress, prevention

## Abstract

Introduction: The impact of the COVID-19 pandemic on suicide risk has been documented during the acute phase, but less is known about people who attempted suicide during the post-acute period. This study investigates how adults who attempted suicide during the post-acute pandemic period (2021–2023) understood COVID-19’s role in their attempt. Method: We analyzed interview data from 329 adults (59% female; 41% male), enrolled following a recent suicide attempt between 2021 and 2023. Participants were asked about the general impact of COVID-19 on their lives and then specifically about whether stress related to COVID-19 was a primary reason for their attempt or contributed to their suicidal thoughts. Results: When asked about their recent attempt, 11% of participants identified stress related to COVID-19 as the primary reason for their attempt, and an additional 23% indicated it contributed to their suicidal thoughts. When describing general impacts, participants reported effects across multiple domains: social isolation, physical health concerns, mental health impacts, and economic effects. Discussion: The attribution of suicide attempts to COVID-19-related stress during the post-acute period highlights the extended impact of public health crises on vulnerable individuals. These findings emphasize the need for sustained, integrated medical and mental healthcare following such crises.

## 1. Introduction

The COVID-19 pandemic prompted unprecedented public health measures worldwide. Social distancing, restrictions on gatherings, and increased reliance on digital technologies were implemented to control viral spread [1,2,3]. While these measures helped contain the virus, they also led to significant changes in how people lived, worked, and maintained relationships. Of particular concern was the impact of these changes on mental health, especially among vulnerable individuals.

During the acute phase of the COVID-19 pandemic (March to December 2020), characterized by peak infection rates, strict public health measures, and widespread restrictions, early research documented increased psychological distress, with particular attention to social isolation and disrupted access to support systems [4]. Studies identified increases in some key factors associated with suicidal behavior, including experiences of thwarted belongingness, economic instability, and barriers to healthcare access [5]. However, as the pandemic evolved, a critical question emerged: How would people understand the role of pandemic-related stress in their mental health and suicide risk over time?

By 2021, the landscape of the pandemic had shifted significantly in the United States. Serious COVID-19 infection rates and most noticeable public health measures (e.g., social distancing, masking, reduced social gathering) gradually decreased as vaccines became widely available. In this study, the post-acute phase is defined as the period following the widespread vaccine availability and the lifting of most restrictions, when infection rates and public health measures had significantly decreased (2021–2023). Previous research emphasizes that the effects of the pandemic on mental health and suicide risk are complex and unfold over time [6], and while research suggests that the pandemic has had lasting effects on mental health [7,8], the extent and nature of these impacts remain unclear. Qualitative investigations of pandemic experiences have revealed how individuals make meaning of pandemic-related disruptions over time, though most of this work focused on general populations rather than those at highest suicide risk [9]. In particular, little is known about how people who attempted suicide during this post-acute period understood the role of pandemic-related stress in their attempts, or how they described the impact of COVID-19 on their lives. Understanding how people who attempted suicide during this later period understood pandemic-related stress’s role in their attempts can inform crisis response planning for future public health emergencies.

During the COVID-19 pandemic from 2020 to 2023, the prevalence of suicide attempts varied considerably across countries [10]. A recent meta-analysis further reported increases in suicidal ideation and suicide attempts during the pandemic, even as suicide mortality rates remained stable [11]. Research has also suggested differing rates of suicidal ideation during the COVID-19 pandemic compared to the pre-pandemic period. Some studies have reported a decrease or stabilization in suicide rates [12], while others have observed an increase in suicidal thoughts and distress [13,14,15,16]. However, most of this research focused on the acute phase of the pandemic, leaving questions about the post-acute period largely unexplored.

The present study addresses this gap by examining how adults who attempted suicide between 2021 and 2023 understood stress related to the role of COVID-19 in their attempts and described the pandemic’s impact on their lives. This timing is particularly significant as it represents a period when most acute pandemic pressures had eased. We sought to understand both how participants described various impacts of COVID-19 on their lives and whether they identified stress related to COVID-19 as playing a role specifically at the time of their suicide attempts.

While most studies have quantitatively examined associations between pandemic conditions and suicide risk [17], we sought to understand how individuals themselves understood the role of pandemic-related stress in their attempts. Our focus on the post-acute pandemic period provides insight into how people made sense of the influence of COVID-19 even after many restrictions had eased. By collecting data shortly after suicide attempts, we minimize the potential for retrospective bias while capturing fresh insights into how people understood pandemic-related stress’s role in their suicidal behavior during this distinct period.

## 2. Materials and Methods

### 2.1. Participants

Participants were drawn from a sample of adults with a recent suicide attempt who were recruited to a randomized controlled trial of the Attempted Suicide Short Intervention Program (ASSIP) conducted at the University of Rochester across Upstate New York. This qualitative content analysis uses only baseline assessments collected prior to randomization and intervention. The study sample comprised 329 adults (*M* age = 34.95, *SD* = 14.86) with a recent suicide attempt who were recruited for the parent study across three sites. Data on COVID-19 items were collected over an approximate 2½ year period (April 2021–September 2023), providing a range of perspectives across different time points in the post-acute pandemic period. Most participants (*n* = 233, 70.8%) completed assessments in the present study within 7 days of their suicide attempt (*M* = 7.7 days following attempt, *SD* = 10.18, range = 0–62). Participants reported a mean of 3.03 (*SD* = 7.67, median = 1) lifetime suicide attempts prior to the index attempt. Because this work is secondary data analysis, data collection was not guided by theoretical saturation criteria and the sampling strategy was not purposive. However, the size and heterogeneity of the sample allowed for the inclusion of a broad range of diverse experiences.

Participants were predominantly female (sex at birth: 59.3%; 40.7% male), identified as women (54.6%; 39.1% men, 5.4% gender minorities, 0.9% declined to disclose), White (73.5%; 13.1% African American, 2.4% Asian, 1.5% American Indian, 7.6% multiracial, 1.8% declined), and non-Latino (87.8%; 12.2% Latino). Inclusion criteria were: (1) 18+ years old, (2) presence of a suicide attempt within the past 60 days (i.e., self-reported suicide attempt with intent to die using a validated item; [18]), and (3) provided permission to contact at least one person if needed to promote participant safety or to reach the participant for follow-up. Exclusion criteria were: (1) presence of psychotic symptoms or other factors that precluded ability to consent or complete the baseline assessment, (2) inability to communicate in English, and (3) resided outside of New York State residence outside NYS during the study intervention period. Potential participants were screened for eligibility either in person (in emergency departments or inpatient psychiatric units) or by phone/videoconference if they were not currently hospitalized.

### 2.2. Procedures

Participants completed assessments in person during their inpatient or emergency department stay, or over the phone/videoconference if not hospitalized at the time of baseline assessment. Research staff took detailed notes during interviews rather than audio recording or capturing responses verbatim; all quoted material in this paper represents these interview notes. All assessments were administered by trained research staff following standardized protocols, including anonymization of the excerpts. Each assessment session was designed to minimize participant burden while ensuring comprehensive data collection. Study staff followed an Institutional Review Board approved risk management protocol and received supervision from investigators on all clinical interviews. Informed consent was obtained from all subjects involved in the study.

### 2.3. Measures

Three items about COVID-19, drawn from previous research on COVID-19 stress [19], were administered in a specific sequence. First, participants were asked an open-ended question about general impact (“In what way(s) has COVID-19 impacted you personally?” followed by, “For example, with respect to your work, your family, your plans, your physical and mental health, or anything else?”). Second, the interview transitioned to focus specifically on the suicide attempt (suicide-specific; “Now I’m going to ask specifically about the time when you attempted suicide.”). Two binary (yes/no) questions were asked to assess whether COVID-19 related stress was considered a primary reason for their suicide attempt (“Was stress related to COVID-19 a primary reason for your suicide attempt?”) and/or a contributing factor (contributed; “Do you think stress related to COVID-19 contributed to your suicidal thoughts at the time of your attempt?”) to suicidal ideation.

### 2.4. Analysis

The study was conducted in accordance with a qualitative research design, applying qualitative content analysis using Atlas.ti 25 software. Qualitative content analysis is a research method for generating reproducible and valid inferences from data in their context, with the purpose of providing new insights, a presentation of facts or a practical guide for action. We used an inductive approach where responses are open-coded directly from the data rather than being predetermined based on existing theory or frameworks [20]. This approach was appropriate for our study because we sought to understand how participants themselves described and made sense of the impact of COVID-19, without imposing preconceived categories. The outcome of the analysis is categories describing the phenomenon and enhancing the understanding of the data. The method is well-suited to analyzing data on the multifaceted, sensitive phenomena characteristic of nursing, psychiatry and public health studies [20].

To ensure comprehensive data analysis and prevent fragmentation, the unit of analysis was defined as the complete response to each question, as recorded in the document for each participant. This allowed for a holistic approach, aligning with the guidelines for conducting content analysis [21]. The first author developed initial codes through repeated reading of responses and preliminary coding attempts. Responses were open coded, using an inductive, exploratory approach, allowing the development of codes directly from the data and better alignment to possible new factors. Multiple codes were applied when appropriate to capture the complexity of responses. These initial codes were refined through several rounds of review and revision in collaboration with the research team during two joint meetings, allowing for triangulation of perspectives. Each iteration involved verification through testing the codes against new segments of data, revising code definitions, and creating interpretative code categories [20,21]. The research team included members with clinical, research, and lived experience perspectives, bringing diverse viewpoints to the interpretation process. To ensure systematic analysis, codes were dichotomized during reporting, with each code counted once per participant to avoid duplication. Frequencies for all 329 participants were imported into Microsoft Excel (Excel 2021 for Windows) for the calculation of the percentage of participants per group, code, and categories.

Based on their responses to the suicide-specific binary items on the COVID-19 questionnaire, participants were categorized into one of three groups: Group 1 (COVID-19 Primary) included those who identified stress related to COVID-19 as the primary reason for their attempt (i.e., said yes to the first binary item); Group 2 (COVID-19 Contributed) included those who indicated it contributed to their suicidal thoughts at the time but was not the primary reason; (i.e., said no to the first but yes to the second binary item); Group 3 (COVID-19 No role) included those who reported no perceived impact of COVID-19-related stress at the time of attempt (i.e., said no to both binary items). While these data were collected during the post-acute pandemic period (2021–2023), when many acute pandemic pressures had eased, our questions asked about past impacts rather than current experiences.

The research team reached consensus on code structure and categories through regular discussions that included consolidating codes, clarifying terminology, and reviewing code placement. All coding decisions were documented and reviewed by multiple team members to ensure consistency and reliability. This iterative process generated 794 quotations across 18 codes grouped into 7 categories. In reporting results, we followed the Standards for Reporting Qualitative Research—SRQR [22]. Quotes from the original data (marked with the group, participant, and quotation number) are selected from different participants.

## 3. Results

### 3.1. COVID-19 as a Primary or Contributing Factor for Suicide Risk

Eleven percent of participants indicated that stress related to COVID-19 was a primary reason for their attempt (Group 1). An additional 23% (who had said no to the primary reason question) reported that while not primary, stress related to COVID-19 contributed to their suicidal thoughts at the time of their attempt (Group 2). The remaining 66% indicated that COVID-19-related stress played neither a primary nor a contributing role (Group 3).

### 3.2. Descriptions of COVID-19’s Impact

When asked the broader question about how COVID-19 had impacted them personally, participants described various effects across multiple domains of their lives during the pandemic period. Their descriptions revealed distinct patterns across the three groups. Table 1 summarizes the qualitative codes and descriptive statistics of the frequencies of these codes across groups.

#### 3.2.1. COVID-19 Primary (11%)

Those who identified stress related to COVID-19 as a primary reason for their attempt (Group 1: COVID-19 Primary) described impacts involving social isolation, work, and mental health. They reported experiences of social isolation and loneliness, work overload, unemployment, or challenges finding employment during the pandemic period. They described limited access to mental health services and feelings of helplessness. Their accounts included tensions in relationships related to recovering from COVID-19 and disagreements over COVID-19 measures and vaccination. A significant theme in this group was a sense of responsibility about infection transmission. An example quote of this theme is “Her responsibility that others got sick from Covid-19”, indicating a participant’s explicit distress at the time of suicide attempt.

#### 3.2.2. COVID-19 Contributed (23%)

Participants who indicated COVID-19-related stress contributed to but was not primary in their attempt (Group 2: COVID-19 Contributed) described similar impacts. They emphasized missed social opportunities and job losses. They described effects of border closures, suspended social activities, and quarantine measures. Their accounts included experiences of social isolation and loneliness, compounded by both fear of infection and experiences of contracting COVID-19.

#### 3.2.3. COVID-19 No Role (66%)

Those who indicated COVID-19-related stress played no role in their attempt (Group 3: COVID-19 No role) generally described fewer or less severe pandemic impacts. Some indicated feeling largely unaffected. An example quote for this group is “My life hasn’t changed besides having to wear a mask.” Some described only short-term effects, for example “Definitely affected the mental health, especially in the beginning”, or indicated that while they experienced isolation, it did not worsen pre-existing feelings of loneliness: “It isolated me, but I already was pretty isolated before COVID.”

### 3.3. Impact Domains Described by Participants

#### 3.3.1. Social Connection and Isolation

Social isolation was the most frequently reported impact (68% of the total sample). Participants described reduced opportunities for social interaction due to social distancing measures, closure of support groups and mental health services, shift to remote work or study, quarantine requirements, and suspended recreational activities. Some, particularly in Group 1 (COVID-19 Primary) and in Group 2 (COVID-19 Contributed), described isolation stemming from fear of close interpersonal contact or crowded settings. For example, one subject described the following: “Father has spots on his lungs, COPD, and asthma, family isolated extra to protect him.” This protective isolation was associated with “worsening depression because of isolating”, as described by another subject. Participants described not only a “lack of social activities” but also a persistent reluctance to go anywhere due to fear of infection. This prolonged isolation, where “day-to-day life became so secluded, and that we couldn’t do much”, was perceived as leading to experiences such as “forgot how to socialize with people.”

#### 3.3.2. Economic and Occupational Effects

In describing the impact of COVID-19, 44% of participants reported work and economic effects, with higher rates in Groups 1 and 2 (63% each). Participants described job losses, reduced income, and difficulties finding employment during the pandemic period. Example quotes for this category are “worked at […], quit because it wasn’t safe”, “Quit job to home school boys”, and “no income with tons of bills”. Some described subsequent regret about employment decisions: “I also withdrew from University […] which I regret. I was making rash decisions.”

#### 3.3.3. Mental Health

Mental health impacts were reported by 45% of participants across all groups in response to the general impact question. They described experiences of social anxiety, depression, sleep disturbances, and panic attacks during the pandemic period. Example quotes for this category: “Devastating to mental health”, and “in hospital with Covid and having depression” illustrate how physical and mental health experiences overlapped. Another participant shared, “son went to camp, though exposed to COVID-19”, and that the main stressor during the time of her suicide attempt was “her responsibility that others got sick from COVID-19.” Intense feelings of guilt and self-blame demonstrate how pandemic-related moral distress and perceived responsibility for others’ well-being contributed to participants’ psychological strain. Persistent fear of transmitting the virus to others highlights how concerns for others’ health intensified stress and anxiety: “My dad is 78 and has diabetes and had open heart surgery. My dad is the most important person to me and COVID-19 added a lot of stress because I was always worrying about him.”

#### 3.3.4. Physical Health

When asked about the impact of COVID-19, 47% of participants described physical health effects, including both experiences with COVID-19 infection and health-related anxiety. Descriptions of significant concern about transmission to others are examples for this category: “Worried about getting covid 19 because of sickle cell anemia. Did not go out 8 months.” Prolonged illness and isolation disrupted key areas of life after the acute phase of the pandemic: “I had contracted COVID for a month. I was isolated in my room. It affected my job and my school year for senior year.”

#### 3.3.5. Family and Relationship Dynamics

Family and relationship impacts were described by 21% of participants, including conflicts over COVID-19 precautions, as captured in an example quote “Constantly arguing that COVID-19 is real. Stopped talking with sister.” Participants described changes in family dynamics, for example “Changed family dynamic due to everyone being at home and working from home”, as well as other stressful life events such as the “end of a relationship.”

#### 3.3.6. Healthcare Access

Healthcare access impacts were reported by 14% of participants, with higher rates in Groups 1 and 2. Many participants described impacts of service interruptions, for example: “Got turned away from crisis clinic […]. Felt unworthy of getting help”, or “difficulty going to […] medical appointments […]. I ended up just using 211.” Additional participants highlighted how the pandemic disrupted essential care and continuity of treatment, stating, “couldn’t get my medications because the supply chains broke down,” “cancel surgeries,” and “stopped seeing therapist and doctors.” These accounts illustrate the compounded impact of limited healthcare access, medication shortages, and canceled treatments on individuals already experiencing psychological distress.

## 4. Discussion

This study examined how adults who attempted suicide during the post-acute pandemic period understood the role of COVID-19-related stress in their suicide attempts and described the pandemic’s impact on their lives.

Our findings represent some of the first evidence about how people who attempted suicide during the post-acute pandemic period understood the role of COVID-19 in their attempts. While existing research has documented associations between the acute pandemic phase and increased suicidal ideation [13,14,15], our study reveals that even after many restrictions had lifted and infection rates had declined, a substantial proportion of people attempting suicide identified pandemic-related stress as relevant to their attempt. Additionally, when asked about the general impact of COVID-19, participants described multiple ways the pandemic had affected their lives during this period.

Social isolation was a prominent theme, with participants describing circumstances that affected their social connections. Findings that physical distancing measures can impact loneliness or social isolation due to the limitations they impose on in-person social contact are consistent with previous research [23]. The unique situation of physical distancing created an experience of loneliness distinct from our prior understanding of loneliness, in which individuals miss contact that involves the physical presence of others, resulting in heightened stress [24]. Our findings clearly indicate that social isolation and loneliness represent the most common stressors among adults who attempted suicide, with physical distancing potentially contributing significantly to feelings of loneliness and to vulnerability to suicide [4,25].

Our qualitative findings indicate that participants did not describe loneliness merely as the absence of social contact or physical proximity, but as a state of diminished, disrupted, or unsatisfactory social practices that persisted over an extended period. Loneliness is a recurrent theme preceding suicide attempts [26]. It represents a core component of thwarted belongingness, one of the central dimensions in the development of suicidal behavior within the Interpersonal Theory of Suicide [27]. Our study suggests that the temporal aspect of loneliness is also important, as it represents a source of stress arising from its prolonged duration, which is often overlooked [28]. Many participants felt that even after pandemic restrictions were lifted, the social world from which they expected emotional or practical support remained diminished and fragile. This suggests that among vulnerable groups, prolonged social disengagement may lead to a slow or incomplete re-establishment of everyday social practices.

Even when social disengagement was perceived as a positive aspect of the pandemic that relieved individuals from demanding social interactions [29], the positive dimensions identified in our study were clearly ambivalent. The temporary relief from challenging everyday social situations did not lead to a lasting improvement in participants’ personal circumstances, which continued to be dominated by feelings of loneliness.

An additional tension, also noted in previous research, such as the conflict between care and deprivation, where social distancing was described as an act of care toward others, yet at the same time felt as abandonment and emotional disintegration [23], was reflected in our findings. Additionally, we identified a case in which moral responsibility for the potential transmission of infection was perceived as the primary stressor at the time of the suicide attempt. In contrast to earlier qualitative research, our participants did not describe COVID-19 as an obstacle to suicide attempt, nor did they express suicidal intent or feasibility related to infection itself [29].

Participants further reported work and economic impacts, from job losses to changes in working conditions. Their accounts included physical health experiences, challenges accessing healthcare, and strains in family relationships. In preventing suicide attempts and deaths during and after significant public health crises, domestic conflict and violence, financial loss, anxiety and depression, and pre-existing mental illnesses [30] should be considered even after major restrictions are lifted. Participants in our research described these various impacts when asked about COVID-19, even during this post-acute period, which suggests complex ways in which they understood the pandemic as having affected their lives.

These findings advance our understanding of how adults who attempted suicide perceived COVID-19 related circumstances. While acute-phase research emphasized immediate stressors like infection fear or restriction-related distress, our findings show that even after many immediate pressures had resolved, some people identified pandemic-related stress as playing a role in their attempt. This has important implications for both research and clinical practice.

The timing of data collection during the post-acute period offered both methodological advantages and limitations. Our study captured how people understood the role of COVID-19 in their suicide attempts during a period when many restrictions had eased, but we cannot compare these attributions to those during the acute crisis phase. Additionally, because we asked about past impacts rather than current experiences, we cannot draw conclusions about whether described impacts were ongoing at the time of interview. While our note-based data collection captured key themes, the lack of verbatim transcripts may have missed nuances in how participants expressed their experiences. While all interviewers followed standardized protocols and received regular supervision, individual variation in note-taking emphasis and detail is an inherent limitation of this approach. The parent study’s location in a single region (Western New York) limits generalizability to broader geographic and cultural contexts.

Looking forward, the finding that some people attributed their suicide attempts to COVID-19-related stress during the post-acute period suggests several directions for research and practice. Future studies might examine how different populations understand COVID-19 circumstances across various post-acute contexts. Additionally, research comparing such attributions across different regions and populations could provide valuable insights for crisis response planning.

This study provides evidence that during 2021–2023, some people attempting suicide identified stress related to COVID-19 as playing a role in their attempts, while also describing various ways the pandemic had impacted their lives. These findings underscore the importance of maintaining enhanced mental health support systems well into post-crisis periods and suggest a need to better understand how people make sense of public health emergencies’ influence on their mental health over time. As we continue to learn from the COVID-19 experience, these insights can help inform more comprehensive approaches to supporting vulnerable populations during future public health crises.

## 5. Conclusions

That some people attributed their suicide attempts to COVID-19-related stress even during the post-acute period has important implications for global crisis response. These findings suggest the need for sustained support well beyond the acute phase of public health emergencies. Future research should examine how different populations understand public health crises’ influence on suicide risk across various post-acute contexts, informing more comprehensive approaches to protecting vulnerable populations during future crises.

## Figures and Tables

**Table 1:** Number and percentage of participants per group, code, and code category.

Impact of COVID-19: Overarching Categories and Detailed Codes **	Group 1 *: COVID-19 Primary (*N* = 35; 10.6%)		Group 2 *: COVID-19 Contributed (*N* = 75; 22.7%)		Group 3 *: COVID-19 No Role (*N* = 219; 66.5%)		Total (*N* = 329; 100%)	
**Social isolation and loneliness**	**28**	**80%**	**62**	**83%**	**134**	**61%**	**224**	**68%**
Feeling isolated and lonely	19	54%	47	63%	97	44%		
Missed social opportunities	9	26%	15	20%	37	17%		
**Work and economic stressors**	**22**	**63%**	**47**	**63%**	**76**	**35%**	**145**	**44%**
Work or academic stress	8	23%	21	28%	39	18%		
Unemployment	9	26%	17	23%	29	13%		
Financial hardship	5	14%	9	12%	8	4%		
**Mental health and psychological distress**	**21**	**60%**	**44**	**59%**	**84**	**38%**	**149**	**45%**
Exacerbation of mental health conditions	12	34%	21	28%	52	24%		
Psychological distress	9	26%	17	23%	30	14%		
Addiciton	0	0%	6	8%	2	1%		
**Physical health**	**19**	**54%**	**38**	**51%**	**96**	**44%**	**153**	**47%**
Getting COVID-19	7	20%	16	21%	47	21%		
Worrying about getting or spreading it	7	20%	14	19%	38	17%		
Physical health issues	5	14%	8	11%	11	5%		
**Family and relationship problems**	**9**	**26%**	**24**	**32%**	**37**	**17%**	**70**	**21%**
Family issues	4	11%	9	12%	20	9%		
Relationships stress	5	14%	7	9%	3	1%		
Somebody close died	2	6%	4	5%	11	5%		
Ending of a partner relationship	2	6%	4	5%	3	1%		
**Access to healthcare and support**	**10**	**29%**	**15**	**20%**	**21**	**10%**	**46**	**14%**
Access to support groups, mental health and other services	7	20%	10	13%	17	8%		
Access to medical services	3	9%	5	7%	4	2%		
**Positive aspects**	**2**	**6%**	**1**	**1%**	**19**	**9%**	**22**	**7%**
Positive aspects of COVID-19 pandemics	2	6%	1	1%	19	9%		

* Group 1 (COVID-19 perceived as a primary reason for suicide attempt), Group 2 (COVID-19 perceived as contributing to suicidal thoughts at the time of suicide attempt), and Group 3 (COVID-19 had no impact on suicide attempt). ** Data for overarching categories are shown in bold.

## Data Availability

The data supporting the findings of this study are available upon request from the second author (A.P.).
